# Supplementary material for: The effect of HIV status on the frequency and severity of acute respiratory illness
Source: PLoS One. 2020 May 29;15(5):e0232977. doi: 10.1371/journal.pone.0232977 (PMC7259631; doi:10.1371/journal.pone.0232977)
Supplement: S1 Data — (PDF) [file pone.0232977.s001.pdf]

Version 1.4: 8th August 2016.

Acute Respiratory Tract Illness in an HIV infected population with a high uptake of antiretroviral therapy.

RFH R&D Reference 9065

UCL Reference 14/0369

IRAS 155055

Study Protocol

Royal Free Hospital

University College London

Background and rationale for this study

Globally, an estimated 34 million people are living with HIV infection, of which there are an estimated 98,000 in the UK. The use of antiretroviral therapy (ART) has resulted in a significant reduction in severe disease and death in HIV infected individuals.[1] Opportunistic respiratory illnesses such as *Pneumocystis jirovecii* pneumonia (PCP), which were previously common and often fatal, are now rare. Bacterial pneumonias and tuberculosis are also less frequent. Survival from a diagnosis of HIV in resource-rich environments is now predicted to be close to that of background populations.[2] The ageing HIV population is not protected from conditions typically associated with older age. In fact the high rates of smoking reported to be associated with HIV mean that the risk of non-communicable illness such as Obstructive Lung Disease may, if anything, be increased.[3] However, it is uncertain whether this is genuinely the case, or if acute respiratory illness has an impact on subsequent development of long-term respiratory conditions.

This study will aim to contribute to a better understanding of the burden of acute respiratory illness in a UK-based HIV infected population with a high uptake of antiretroviral therapy and to evaluate the possible relationship between these acute events and the development of chronic lung disease.

What is known about rates of respiratory disease amongst HIV infected individuals with access to antiretroviral treatment?

Current evidence suggests that HIV infected populations have very high rates of respiratory symptoms, with up to 63% of individuals reporting chronic respiratory symptoms.[4] This burden of disease appears to be significantly higher than equivalent HIV-uninfected populations, although few studies have directly compared similar HIV infected and uninfected individuals. It is likely that this burden of disease is the result of interplay between known risk factors such as smoking and HIV-specific effects: HIV infected individuals have high rates of smoking of tobacco and other recreational drugs, but in addition, several studies have suggested that HIV infection is an independent risk factor for the development of respiratory disease.[5] If this is the case, this might be mediated by direct effects of the HIV virus, a disordered immune response or the result of more frequent or more persistent respiratory infections.

Rates of respiratory tract infection in HIV infected populations with access to anti-retroviral treatment.

HIV-infected individuals have been known to experience high rates of acute respiratory illness since the start of the HIV epidemic. The epidemiology of respiratory illness prior to the use of ART was detailed by the Pulmonary Complications of HIV Infection Study, which followed 1,353 HIV infected and 183 HIV uninfected individuals in the USA between 1988 and 1994.[6] The most prevalent respiratory diseases in this study were upper respiratory tract infections (which occurred at a rate of 42.3/100 person years in those with HIV

infection compared to 29.4 per 100 person-years in those without) and bronchitis (which was twice as common in those with HIV infection). Although less prevalent, bacterial pneumonia was six times more common in the HIV infected.

The use of effective antiretroviral therapy has changed the epidemiology of respiratory disease in those with HIV infection. Several large observational cohorts and population based registry studies have provided evidence regarding the rates of respiratory illness since the development of ART. These suggest that an increased frequency of acute respiratory illness compared to HIV uninfected individuals persists. The Multi Centre AIDS Cohort Study has recruited men in the US since 1984.[7] Data from this study since 1996 (when ART became available) report an OR of 1.51 for bronchitis; 1.46 for sinusitis and 4.14 for pneumonia compared to the HIV uninfected participants in the study. The Women's Interagency HIV study, which has recruited women in the US since 1994, reported an OR of 2.17 for sinusitis ($p<0.001$), 1.46 for acute bronchitis ($p= 0.22$) and 9.55 ($p <0.001$) for pneumonia since the development of ART. The Veterans Aging Cohort Study in the USA has used registry data to evaluate rates of disease and reports that pneumonia is around 5 times more common in those with HIV infection compared to those without.[8] Higher CD_4 count and lower plasma HIV viral load were reported to be protective against bacterial pneumonia in this study.

Despite the high frequency of acute non-pneumonic respiratory illness, few studies have documented the epidemiology or underlying microbial causes in a systematic manner. A case series from Montreal, using molecular diagnostics, found that of 50 HIV infected patients with fever and symptoms consistent with respiratory tract infection, viruses accounted for 64% of these illnesses.[9] Not only might these acute respiratory illnesses have a short-term impact on individuals, but it is possible that they may affect longer term outcomes too.[10]

Rates of Non-communicable Respiratory Disease

HIV infected populations have higher rates of a range of lung diseases, including COPD, lung cancer, bronchiectasis and pulmonary hypertension.[3] As the most prevalent conditions, Obstructive Lung Disease (primary asthma and COPD) are of particular importance. A number of recent studies have reported the prevalence of OLD in current HIV infected populations; estimates of the proportion of HIV infected populations with obstructive lung disease range from 4.6% to 23% (see Tables 1(a) and 1(b)). Few studies have directly compared HIV infected and uninfected participants and these do not all find HIV infection to be an independent risk factor for the development of obstructive lung disease or the presence of chronic respiratory symptoms. Analysis of a cohort of current and previous injection drug users in Baltimore has suggested that HIV viral load (but not CD_4 count) is an independent predictor of accelerated lung function decline,[11] and interestingly higher HIV viral load was also reported to be associated with a diagnosis of COPD in the Veterans Aging Cohort Study data.[8]

Table 1(a) Studies comparing rates of spirometric abnormality in HIV infected individuals with access to antiretroviral therapy compared to uninfected individuals.

First Author	Date	Number of participants	Site of study	Method of assessment	Rate of OLD/COPD in HIV positive participants	Rate of OLD/ COPD in HIV negative participants
**Drummond**[12]	2010	686 HIV negative 288 HIV positive	Baltimore, USA	Pre-bronchodilator spirometry	16%	16%
**Madeddu**[13]	2013	111 HIV positive 65 HIV negative	Sardinia	Post-bronchodilator spirometry and TLC	23.4%	7.7%
**Crothers**[14]	2013	300 HIV positive 289 HIV negative	USA various sites	Post-bronchodilator spirometry and DLCO	18%	16%
**Campo**[15]	2014	180 HIV positive 160 HIV negative	USA, various sites.	Post-bronchodilator spirometry, lung volmes and DLCO	19%	20%

Table 1(b) Studies comparing rates of diagnosed COPD in HIV infected and uninfected individuals using registry data.

Author	Date	Population	Site of Study	Method of identification	COPD prevalence HIV positive	COPD prevalence HIV negative
Crothers[8]	2011	VACS Cohort: 33,420 HIV infected; 66,840 matched controls	USA, Veterans Affairs Health service	ICD-9 codes	4.6%	4%
Kendall[16]	2014	14,005 HIV infected, 71,410 general population	Ontario, Canada.	ICD-9 codes	8.33%	5.33%

Other studies underway in this field

A search of clinicaltrials.gov reveals several studies examining the rates of obstructive lung disease and factors contributing to this. In addition, a pulmonary sub-study of the START trial (a large multi-centre randomised controlled trial of delayed vs immediate antiretroviral therapy for HIV infected individuals with CD4 counts of over 500) will evaluate rates of lung function decline and the influence of ART in their study subjects. However, to our knowledge there are no cohort studies underway looking at the effect of acute respiratory illnesses in this population or prospectively evaluating pathogens associated with these illnesses.

Measurement of Health-Related Quality of Life

In order to assess the impact of illness and the acceptability of treatments it is essential to measure health-related quality of life. This has become increasingly important in the field of HIV research as improvements in life expectancy with treatment make an evaluation of quality of life central to HIV care.[17,18]

The assessment of health-related quality of life can utilise generic measures such as the Euro-Qol 5D;[19] disease specific instruments such as the Medical Outcomes Study-HIV (MOS-HIV) and the Functional Assessment of HIV infection (FAHI) [20] or system-specific instruments such as (for respiratory illness) the St Georges Respiratory Questionnaire.[21] The combination of generic and disease or symptom specific instruments can add significantly to the information gained by the use of any one tool.

In the context of HIV infection, quality of life assessments have been extensively utilised and have been shown to be capable of identifying the effect of HIV infection on quality of life and changes with treatment. [22,23] To assess the burden of respiratory disease the St Georges Respiratory questionnaire was originally developed to measure quality of life in COPD, but has subsequently been used in many different respiratory conditions. [24]

Less is known about the effect of respiratory disease on quality of life in HIV infected individuals. Two studies have used the St George's Respiratory Questionnaire to assess respiratory symptoms in HIV infected individuals[25,26] and these have demonstrated high rates of symptoms. Analysis of quality of life data in the ALIVE cohort of current or former injection drug users in Baltimore demonstrates that both HIV and Obstructive Lung Disease are associated with reduced health related quality of life.[27] However, these cross-sectional studies have not specifically evaluated the effect on quality of life of incident *acute* respiratory illness.

This study we will use the Euro-Qol 5D-5L as a generic measure of health outcomes and the St George's Respiratory Questionnaire (SGRQ) to assess respiratory specific health status. The SGRQ takes about 15 minutes to compete and the EQ5D takes around 5 minutes to complete.

Measurement of Healthcare Resource Utilisation

Acute respiratory illnesses are responsible for significant healthcare resource utilisation in the general population: for instance, over 25% of the population visit their GP each year regarding a respiratory infection.[28] Rates of GP consultation for respiratory tract infections range from 70.5/1000 per annum for acute sore throat; 15.2/1000 for rhino-sinusitis and 18.1/1000 for acute bronchitis.[29] A UK prospective observational study based in primary care found a rate of consultation for lower respiratory tract infections of 53/1000 per annum in previously well individuals between the ages of 40-59 (the age cohort most closely corresponding to our study population).[30]A UK national cohort study (which reported rates of acute respiratory illness per influenza season) found rates of acute respiratory tract illness of 44 per 100 person-seasons in those with no serological evidence of influenza and 69 per 100 person-seasons in the 18% of people with serological evidence of influenza.[31] Acute respiratory tract illnesses are therefore associated with significant direct and indirect costs to healthcare systems and society – for instance it is estimated that pneumonia in working age adults in the US is associated with a cost of $10 billion.[32]

Acute respiratory illnesses are common and associated with significant healthcare utilisation, so if HIV infection increases their frequency or severity this could significantly impact on healthcare resource utilisation. Although several studies have demonstrated high rates of respiratory symptoms and disease amongst HIV infected individuals (as discussed above), these studies have not included an assessment of the cost associated with these comorbidities. To our knowledge there has been no published assessment of the impact of acute respiratory illness on healthcare resource utilisation in HIV infected individuals. An

understanding of this is essential for the effective evaluation of the cost effectiveness of interventions to improve respiratory health and the planning of healthcare services. We will therefore record all contact with healthcare services and treatments received by study subjects relating to acute respiratory tract illnesses. This will include utilisation of primary care services (both General Practitioner and Emergency Department visits), hospital admissions, and contact with the trial investigators. We will also collect data regarding time off work or usual activities caused by these illnesses to assess the impact on individuals and their productivity.

Study objectives

Hypothesis: HIV infected individuals experience an increased incidence of acute respiratory tract illness despite the use of antiretroviral therapy. This contributes to increased respiratory symptoms and may be associated with increased rates of obstructive lung disease in the long-term.

Aim: To determine the influence of HIV infection on the frequency and impact of acute respiratory tract illness.

Objectives:

1. To follow a prospective cohort of individuals with and without HIV infection over a 1 year period to determine the frequency of acute upper and lower respiratory tract illness.

2. To document the duration of symptoms of acute respiratory tract illness using self-completed diary cards.

3. To document the impact on health-related quality of life of acute respiratory illnesses using the St Georges Respiratory Questionnaire and the EuroQoL-5D.

4. To determine the frequency and spectrum of respiratory organisms isolated at baseline and during follow up within the cohort.

5. To determine the healthcare utilisation associated with these illnesses.

6. To determine the relationship between respiratory events and cohort characteristics such as cigarette smoking.

7. To determine the relationship between baseline spirometry and acute respiratory illnesses.

Primary Study Outcome

The annual incidence of acute respiratory illness in HIV infected adults compared to those without HIV infection.

Secondary study outcomes:

1. Duration of symptoms during respiratory tract illness in HIV infected and uninfected (objective 2).

2. health-related quality of life measured by the St Georges Respiratory Questionnaire and EuroQoL-5D (objective 3).

3. Healthcare resource utilisation arising from acute respiratory illness (objective 5).

4. The prevalence of positive microbial isolation at baseline (objective 4).

5. The prevalence of positive microbial isolation during acute respiratory illness (objective 4) and the duration of viral shedding in those with respiratory viral infections.

6. The baseline prevalence of obstructive lung disease (objective 7)

Methods

A: Systematic Review

A systematic review of the literature will be undertaken concerning the rates of respiratory symptoms HIV infected individuals compared to HIV uninfected individuals in the ART era.

Systematic reviews of clinical studies relevant to these questions will be undertaken. As outlined in the Cochrane Handbook and the PRISMA statement,[33,34] review preparation will follow six steps:

1. Formulation of the problem
2. Location and selection of studies
3. Critical appraisal of the studies
4. Collection of the data
5. Analysis and presentation of the results
6. Interpretation of the results.

Search strategies will include both computerised literature search of a number of databases including PubMed/Medline/PreMedline, Scopus, Embase, CINAHL, Google Scholar, Web of Science, BIOSIS, DARE, Global Health and Index to Theses. Search terms will be defined in discussion with a reference librarian with extensive experience in literature searches to ensure capture of all relevant studies. Relevant references from articles/reports identified will also be obtained. We will also conduct hand-searches of key journals, reports and bulletins and website searches of relevant studies.

Completing these reviews will enable a summary of the current evidence base to be produced. These reviews will be registered with the PROSPERO website.

B: Clinical study

This will be a prospective observational study of a cohort of HIV infected and uninfected participants followed up for one year.

Study population

We will recruit a study cohort of HIV infected and uninfected individuals. In total we will recruit 140 individuals with HIV infection and 70 HIV uninfected individuals and follow up these individuals for one year.

HIV infected participants will be invited to participate from the outpatient clinic at the Royal Free Hospital. This hospital has a population of over 2,500 individuals currently attending for HIV care and is the site of numerous previous clinical studies including prospective cohort studies and randomised controlled trials. Staff in the HIV Department are therefore familiar with clinical research, and the environment is supportive of such studies. Past experience and discussion during the design of this study indicates that patients are keen to contribute to an understanding of HIV infection and to improvements in care by participating in research. We are therefore confident that we will be able to recruit a representative cohort from this outpatient population sufficiently rapidly to allow adequate prospective follow up.

The ambulatory service of the HIV Department at the Royal Free has detailed demographic information about the attending population. This allows us a good understanding of our proposed study population. They have a median age of 46 years; 74 % are male, 84% have an undetectable plasma HIV viral load and the median CD4 count is high (at 626 cells/µL). This is therefore an ideal site in which to study respiratory tract illness in a stable, ART-treated, population. An important influence on frequencies of lung disease and respiratory infection will be rates of tobacco and recreational drug smoking. Data collected for an unrelated study in this clinic population suggest there are high rates of smoking with 39% of the current clinic population being current smokers (Capocci, personal communication).

To allow valid comparisons, we will recruit HIV uninfected participants with similar demographic, ethnic and age background to the HIV infected participants in the study. HIV uninfected subjects will be invited to participate in the study by HIV infected subjects and recruited from several other sources (see below).

Recruitment of subjects

HIV-infected participants will be selected from the HIV ambulatory care clinic (Ian Charleson Centre) at the Royal Free Hospital. Patients will be approached by their usual clinical care team during routine appointments. We will randomly select potential participants from clinic lists (3 per clinic) by the use of a random-number generator. Potential study subjects will be approached by their regular doctor during routine consultation if he/she fits inclusion criteria. Patients will be given an information sheet and any questions can be answered by the researchers. Written informed consent will be obtained at this point.

HIV-uninfected participants will be recruited using a variety of methods:

1. HIV infected individuals within the cohort will be asked to nominate three HIV uninfected individuals for possible inclusion in the study. One of these will be randomly selected and approached.

2. Subjects will be recruited from Sexual Health clinics at the Royal Free Hospital (Marlborough centre). Potential participants will be approached by the clinical teams in this clinic. If individuals might be willing to participate in the study they will be given further verbal and written information by study staff and arrangements made for review in a study clinic.

3. Further HIV uninfected participants in the study will be recruited from hospital (non-clinical) staff.

4. If necessary, subjects will also be recruited from local primary care practices; they will be representative of the HIV-infected clinic population on the basis of age, gender and ethnicity. Limited demographic details from individuals who decline to participate in the study will be recorded if they consent to this, as part of study quality assurance.

Participants will be reimbursed for reasonable transportation costs for visits that do not form part of their routine treatment/healthcare.

Following completion of this study, participants will be encouraged remain as part of an on-going cohort study who can then be followed over a longer period to allow longitudinal assessments of respiratory health and change in lung function over longer periods. This would be voluntary and participants can opt not to participate in this further follow up if they wish to do so.

Recruitment into this study

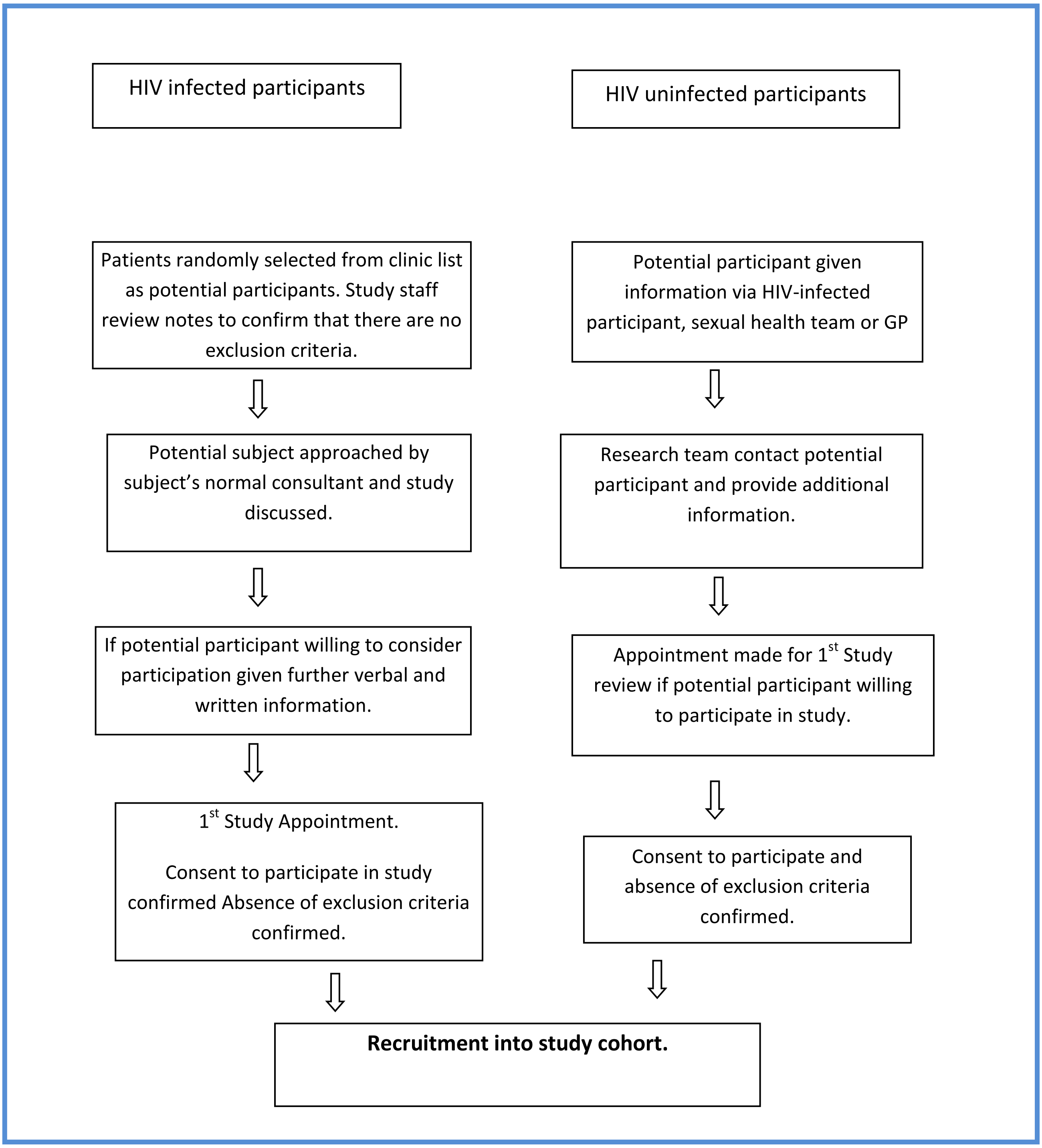

Inclusion and Exclusion Criteria

Inclusion criteria for HIV-infected cohort subjects:

- Willing to participate in study and able to return for review in the event of respiratory tract infections, and to participate for the duration of the study
- 18 years or above

Inclusion criteria for HIV-uninfected participants

- Willing to participate in study and able to return for review in the event of respiratory tract infections, and to participate for the duration of the study
- 18 years or above
- Consent to HIV testing
- Negative HIV test

Exclusion criteria (for both study groups):

- Unable to complete spirometric manoeuvres
- Unable to perform induced sputum collection (e.g. due to bronchospasm)
- Unable to participate for the full duration of the study
- Unable to return for review in the event of respiratory tract infection (for instance those living a long distance from the study site)
- Current significant acute respiratory tract illness such as pulmonary tuberculosis, *Pneumocystis jirovecii* pneumonia

Data collection

Participants in the study will be interviewed at recruitment and then reviewed regularly every 4 months during the study and at all times of acute respiratory illness. Data will be recorded in a proforma and entered into a database under allocated study number. This data will only be accessible by the trial investigators. This is pseudo-anonymised with only the clinical care team having the original code for patient identifiable data. The personal information and link will be stored in a protected encrypted database.

Baseline data collection for all study participants will consist of:

- Demographic details
- HIV and non-HIV related medical history
- History of tobacco and recreational drug use including use of electronic cigarettes
- History of pneumococcal and influenza immunisation
- Medication history
- Social history: employment; educational attainment; young children within household
- For those in the HIV infected cohort: Nadir CD4 count; duration of known HIV infection; highest recorded HIV plasma viral load; HIV tropism (if known); risk for HIV acquisition; ever AIDS diagnosis; plasma CD_4 count and HIV viral load at baseline
- The burden of respiratory symptoms will be assessed by use of the St Georges Respiratory Questionnaire (SGRQ) and general health status by the EuroQoL 5-dimension (EQ-5D). These are well established research tools for evaluating respiratory symptoms and health status[35,36]
- Naso-pharyngeal swab for detection of respiratory viruses
- Spontaneous or induced sputum collection for detection of respiratory viruses and bacteria by means of culture and molecular diagnostic techniques including mycobacterial culture
- Spirometry with bronchodilator reversibility
- Samples (blood and sputum) will be taken for analysis of host immunological response. Where possible samples will be stored for future analyses relevant to this study

Assessment during acute respiratory illnesses will consist of:

- Current respiratory symptoms and health-related quality of life as assessed by EuroQol-5D and EXACT score
- History of relevant exposures: smoking, use of electronic cigarettes, use of recreational drugs, history of pneumococcal and influenza immunisation
- Naso-pharyngeal swab for detection of respiratory viruses
- Spontaneous or induced sputum collection for detection of respiratory viruses and bacteria

- Blood tests for assessments of host immune response
- During acute respiratory illnesses, participants will be asked to complete daily diary cards of their on-going symptoms until these return to baseline to allow analysis of the duration of these acute respiratory illnesses
- Most recent HIV plasma viral load and CD_4 count (for HIV infected participants)

We will use the EXACT-PRO score to evaluate the severity of the acute respiratory illnesses. This can be used as a daily diary completed by participants to allow monitoring of the duration and severity of the acute events.[37] We will arrange to review participants around 2 weeks after the onset of an acute respiratory illness to obtain data regarding the duration and severity of illness.

Table 2: Timing of data collection

	Baseline	4 months	8 Months	12 months	During acute respiratory illness
**Demographic details.**	✓	×	×	×	×
**Collection of details regarding past medical history.**	✓	×	×	×	×
**History of influenza and pneumococcal immunisation**	✓	✓	✓	✓	✓
**Smoking history**	✓	✓	✓	✓	✓
**History of use of other recreational drugs**	✓	✓	✓	✓	✓
**St George's Respiratory Questionnaire**	✓	✓	✓	✓	×
**Euro QoL-5D-5L**	✓	✓	✓	✓	✓
**Spirometry**	✓	×	×	✓	×
**Naso-pharyngeal swab for respiratory virus detection**	✓	×	×	×	✓
**Spontaneous or induced sputum for respiratory virus detection**	✓	×	×	×	✓
**Spontaneous or induced sputum for detection of bacteria**	✓	×	×	×	✓
**Blood tests for measures of host immune response**	✓	×	×	×	✓

Further assessments to be performed as appropriate for some participants:

1. In those participants with a positive result for a respiratory virus (such as influenza) we will ask the participant to return for repeat sampling after 7 days to evaluate the duration of viral shedding. If this second sample remains positive then this test will be repeated weekly until it becomes negative.

2. When this is clinically indicated, participants in the cohort may undergo bronchoscopy to assist in the diagnosis of acute respiratory illness. In this circumstance, broncho-alveolar lavage fluid will also be obtained for microbiological analysis and analysis of host immunological response.

3. Participants in this study, as part of routine treatment/care, may have tests as required by their clinical care team; such tests may include Chest X-Ray, CT chest, lung function tests and echocardiography. Although these tests are routine care and as such fall outside of the research study protocol, we will inform participants of our intention to collect and use these data in the PIS and secure consent for the use of information from such tests in the participant consent form. .

4. Further analysis of a subgroup of the study population will be undertaken by use of exhaled nitric oxide (FeNO) to evaluate this measure of airway inflammation and analysis of aortic pulse wave velocity to assess cardiovascular disease risk. Both of these techniques are non-invasive assessments that have been used in multiple other studies and in clinical practice.[38,39] Some participants will be asked to give urine samples to explore possible biomarkers of lung damage including urine desmosine levels.[40] These samples will be stored for future analysis relevant to this study.

5. In order to further investigate the suggestion that HIV infection is associated with an increased incidence of obstructive sleep apnoea, a subgroup of HIV infected and uninfected individuals will be asked to complete an Epworth Sleepiness Score questionnaire. This is a standard assessment of hyper-somnolence. Those with scores of 10 or more will then be offered sleep studies to exclude the possibility of obstructive sleep apnoea.

6. Samples obtained in this study will be used to evaluate host immune function and burden of microbiological colonisation.

Where possible, samples of body fluids obtained from this study will be stored for subsequent microbiological analysis of analysis of host immune response and evaluation of biomarkers of lung disease**.** These samples will be stored in a secure location in the microbiology department of the Royal Free Hospital. Samples will be stored in pseudo-

anonymised form by study number and access to the study numbers will be restricted to study investigators.

Study Procedures

1. Induced sputum. This will be undertaken at recruitment to the study and at times of acute respiratory illness. Samples will be collected using nebulised 3.5% saline from study subjects who do not spontaneously produce sputum. This will be undertaken in a negative pressure chamber to avoid any chance of transmission of infection during the test. The test takes about 20 minutes. Spirometry will be performed before the procedure and repeated after 5 minutes of sputum induction due to the small risk of inducing bronchospasm by sputum induction. Study subjects known to have asthma will be offered salbutamol prior to the test to reduce the risk of causing wheezing. Subjects with poorly controlled asthma or asthma symptoms at the time of attending will not undergo sputum induction. Although contamination from the upper respiratory tract is clearly possible when using this method, recent publications have confirmed that the use of induced sputum can provide good quality lower respiratory tract samples which allow analysis of populations of bacterial pathogens.[41]

2. Spirometry. This will be performed at recruitment to the study and then repeated each year. Subjects will undergo verbal instruction in the technique, including what the test entails and how they may feel during and after the test. Spirometry will be performed a minimum of 3 times, whilst seated, with appropriate single use one-way filter. FEV1, FVC and peak flow, plus quality of flow loop will be recorded at each attempt. All attempts will be recorded and the best used as study measure. The FEV1 and FVC at two attempts should not differ by ±5%. Coughing during an attempt will render the attempt invalid. Each subject undergoing bronchodilator reversibility will then inhale 2 puffs of salbutamol at 50µg/puff using a metered-dose inhaler with volumatic spacer (unless subject has previously used salbutamol within the preceding 4 hours). Spirometry will be repeated 15 minutes later. The results (including lung age) will be conveyed to the subject at their request. Contra-indications to spirometry consist of: recent pneumothorax or thoracic surgery (within last 3 months), recent myocardial infarction or stroke (within last 1 month), recent abdominal, eye or neurosurgery (within last 2 months), recent perforated tympanic membrane (within last 3 months) Unstable angina, haemoptysis of unknown origin, systolic blood pressure >190mmHg.

Where investigations identify significant clinical abnormalities (for example abnormal spirometry) study participants will be referred to an appropriate medical clinic for follow-up and management. In the majority of cases this will be to the existing dedicated respiratory-HIV clinic. Further follow-up of abnormal results will be undertaken where clinically indicated.

Biological Samples

In the study, blood, sputum and naso-pharyngeal swabs will be collected from patients in accordance with the patient consent form and patient information sheet and shall include all tissue samples or other biological materials and any derivatives, portions, progeny or improvements as well as all patient information and documentation supplied in relation to them.

The sputum and nasopharyngeal swabs will be appropriately sent to the microbiology and virology department for investigation of pathological organisms by culture methods and molecular diagnostic techniques including RT-PCR in accordance with the analytical plan agreed with the Chief Investigator.

The microbiology and virology departments of the Royal Free Hospital will process, store and dispose of the blood, sputum and swab samples in accordance with all applicable legal and regulatory requirements, including the Human Tissue Act 2004 and any amendments thereto.

Samples taken during this study will not be transferred to any party not identified in this protocol and are not to be processed and/or transferred other than in accordance with the patients' consent. After ethics approval for the study has expired, the blood, sputum and naso-pharygneal swab material will be disposed of in accordance with the Human Tissue Act 2004, and any amendments thereto, or transferred to a licensed tissue bank.

Prospective follow up and use of diary cards

Study subjects will be followed for 1 year. Over this time they will be routinely reviewed every four months, thus providing three routine reviews during the study. We will ask subjects to complete the SGRQ and EQ-5D to provide prospective data on respiratory and general health status at each 4-monthly visit. Spirometry will be repeated at the end of the study. Regular review of participants will aid retention in the study.

Between these points of routine review, subjects will be requested to complete weekly diary cards detailing respiratory symptoms throughout the study period. These will ask if subjects have had any symptoms suggestive of acute respiratory tract illness (see below) in the preceding 7 days: To optimise retention in the cohort we will provide diary cards in both printed form and as a website that can be accessed by use of a unique study number to allow submission of information regarding symptoms. The intention of the diary cards is to ensure documentation of all episodes of acute respiratory illness and avoid recall bias that would be inevitable with retrospective documentation of these events. The majority of the time, most participants will simply record “no new symptoms” and this should take less than 5 minutes per week.

Self-reported diary cards have been used successfully in respiratory cohort studies of chronic obstructive lung disease.[42] A balance needs to be struck between the frequency of sampling/completion of cards, the number of areas which they explore and the pragmatism of getting someone to complete these on a regular basis without developing “study fatigue”. The widespread availability of electronic media has enabled mass communication and with appropriate personal safe-guards it is possible for patients and study participants to send information to healthcare workers and researchers on a regular and routine basis.

We have identified a software company with extensive experience in market research including the use of technology to allow responses from patients in the NHS (Formic Solutions) who are able to provide a fully adaptable electronic form that allows uniquely identifiable responses (by study number) that will enable subjects to easily return their diary card information electronically. We will ask subjects to contact a member of the study team if they develop symptoms suggestive of a respiratory tract infection. The study team will be accessible by a dedicated telephone number and email address.

The use of electronic submission of diary card information will allow data to be efficiently collected from study subjects without imposing too great a burden on them.

Following completion of this study, participants will be asked if they are prepared to enter on-going follow up as part of a cohort looking at long-term respiratory health.

Review at times of acute respiratory tract illness

Subjects will document the *acute onset* of cough, blocked or runny nose, fever, breathlessness or chest pain. We will ask subjects to record contact with health services (including primary care and hospital ambulatory care services) with regard to these respiratory symptoms. With study participant's consent, the reported use of healthcare resource will be confirmed by contacting other healthcare providers to verify utilisation.

When study participants develop symptoms suggestive of an acute respiratory tract illness they will be asked to contact the primary investigator and we will review them within 48 hours of symptom onset and obtain samples for analysis. Study subjects will be able to contact the investigating team via a dedicated telephone number, via email, or by visiting the HIV outpatient clinic. These reviews will occur in the Ian Charleson Day Centre (HIV ambulatory care centre) or in the Grove respiratory unit at the Royal Free depending on available capacity. At these times subjects will repeat the assessments of their current health-related quality of life. We will obtain nasopharyngeal swabs for detection of respiratory viruses and sputum samples for both viral and bacterial detection by culture and molecular diagnostic techniques. We will take blood samples for evaluation of host immune response and HIV-specific parameters (e.g. HIV viral load) in HIV infected participants. Subjects will be encouraged to record how for how long their respiratory illness lasts and any contact with healthcare providers by means of daily diary cards during these acute illnesses. Study subjects who attend with symptoms of respiratory tract infection will be managed as per routine clinical guidelines.[43] Further investigations such as additional blood tests and chest radiograph will be obtained when clinically indicated. In order to evaluate the duration of these acute respiratory illnesses, we will ask participants to complete more detailed daily diary cards to record their on-going symptoms until these return to baseline.

Case definitions of acute respiratory illness

The primary end-point of this study will be the frequency of acute respiratory illness (ARI). This will be defined as the acute onset of any of the following symptoms lasting more than 24 hours:

- cough
- sore throat
- blocked or runny nose with or without a sensation of facial pain or pressure
- breathlessness or pain on breathing

In addition we will collect information about the presence of fever, myalgia or sputum production (although fever or myalgia alone will not meet the criteria for ARI), this information will be used to characterise episodes of pneumonia or influenza like illness, but do not form part of the definition of ARI.

Previous studies of acute respiratory illness in the community have used a variety of case definitions of acute respiratory illnesses. For instance, a large community-based observational study in the USA utilised 8 symptoms to define 5 syndromes of acute respiratory tract illness. [44] Much epidemiological work has used simple clinical case definitions to identify acute viral respiratory tract infections but these are primarily designed to track changes over time rather than capture all cases.[45] In addition, there is considerable overlap between the symptoms of different forms of respiratory tract infection and symptoms may change with time making the definition of each event difficult. [46] Despite these problems simple syndromic definitions have been successfully used in community based studies of lower respiratory tract infection in primary care,[47] rates of acute respiratory illness amongst service personnel[48] and population surveillance of acute respiratory disease.[49]The symptoms included in the definition of acute respiratory illness in this study have been chosen to capture all acute respiratory tract illness (including acute rhino-sinusitis) but will not include acute otitis media.

In order to gain an understanding of the patterns of respiratory tract illness, we will use the symptoms reported to subdivide each acute respiratory tract illness as detailed in Table 3. All symptoms refer to the acute onset of symptoms lasting more than 24 hours.

Table 3: Case definitions

	Cough	Blocked or runny nose	Sore Throat	Cough productive of sputum	Breathlessness or pain on breathing	Fever	Myalgia	Change on CXR consistent with infection.
**Acute upper Respiratory Tract Illness**	✓*	✓*	✓*	✗	✗	§	§	✗
**Acute lower Respiratory Tract Illness**	✓**	§	§	✓**	✓**	§	§	✗
**Influenza- Like Illness[50]**	✓	§	§	§	§	✓	✓	§
**Pneumonia[51]**	✓**	§	§	✓**	✓**	§	§	✓

✓ Symptom required for definition
✗ Symptom must not be present
§ Symptom may be present but not required for definition
* Any one of these three symptoms sufficient for definition
** Any one of these three symptoms sufficient for definition

Statistical analysis and minimisation of bias

Statistical analysis of study results will be undertaken to determine the significance of, and differences in, the primary and secondary study outcomes between the study groups. Baseline characteristics of the HIV –infected and HIV uninfected cohorts will be compared by use of the t-test or Wilcoxon rank-sum test for normally and non-normally distributed continuous values. Chi-square tests will be used for categorical values. For the primary analysis, incidence of respiratory infections will be calculated comparing the HIV infected and uninfected populations using univariable and multi-variable Poisson regression analysis. Confounding variables, including time changing factors, will be included in the fully adjusted model based on a detailed analysis plan that pre specifies a priori confounders and other variables with a p value of 0.2 more in the univariate analysis. We will also investigate each variable for effect modification.

We will evaluate possible confounding factors which could cause observed differences between the two cohorts in our study by collecting information on known risk factors for respiratory infection and respiratory illness. These will include: tobacco smoking; use of recreational drugs; occupation; history of pneumococcal or influenza immunisation.

In order to minimise recruitment bias into the study we will select patients attending follow up appointments to be invited to participate without access to clinical records by reviewing patient numbers 2, 4 and 7 for possible invitation to the study. We will collect demographic details of those who decline to participate in the study so that we can evaluate any biases in the study population.

One potential source of bias is that individuals with HIV infection may be more likely to report respiratory tract infections, perhaps because of an awareness of the potential severity of respiratory tract infections in the context of HIV infection. This makes the use of diary cards to prospectively record respiratory symptoms very important, as we hope to record a high proportion of all respiratory tract infections occurring in our population, and which will thus minimise bias in reporting of infections between the two groups. To facilitate this we will create a study email address, there will be a dedicated study mobile phone and we will allow online submission of symptoms on a dedicated website. The use of information technology to send prompts to subjects to complete the diary cards, and online submission of data by subjects, will make this process simpler for study subjects to complete and thus enhance retention in the study.

Calculation of required study size

The primary study outcome will be the number of acute respiratory tract illnesses occurring over a one-year follow up period. The Pulmonary Complications of HIV study, Multicentre AIDS Cohort Study and the Women's Interagency HIV study all found that HIV infected participants had rates of respiratory tract infections approximately 50% higher than in HIV uninfected individuals. We will include upper and lower respiratory tract infections in our primary outcome measure.

The FluWatch study of the epidemiology of influenza in the UK provides information regarding expected numbers of acute respiratory illnesses in our study.[31] Using methodology similar to that proposed in this study (weekly diaries of acute respiratory illnesses) FluWatch reported that 44% of those without serological evidence of influenza infection experienced an acute respiratory illness each influenza season (the study was only conducted during influenza seasons). Based on this we conservatively estimate that at least 44% of the HIV negative participants will have an acute respiratory illness over a 12 month period. Both the Pulmonary Complications of HIV infection study and the Multicentre AIDS cohort study found around a 50% increase in acute respiratory illnesses, so we therefore predict that 68% of the HIV infected individuals will develop an acute respiratory illness over a 12 month period.

We plan to have a 2:1 ratio between HIV infected participants and controls, as we anticipate that the HIV infected participants will be easier to recruit. To have an 80% power to detect this difference (i.e. 68% vs. 45%) with a type 1 error of 5% we would need 119 HIV infected and 60 in the HIV uninfected participants in the cohort. If we assume up to a 20% drop out of subjects from the study then we plan to recruit 140 individuals with HIV infection and 70 individuals without HIV infection.

Study Conduct

The principal investigator and study supervisors will be responsible for study oversight. We will have administrative support from The Joint Research Office Infrastructure from the Royal Free and UCL.

In order to provide formal review of the progress of the study, a Steering Group will be established. This will consist of the research team, 2 patient representatives and a statistician. Although the services of a statistician are not included in the budget for this study, these costs will be met from existing departmental funds. This group will meet prior to study initiation and at a planned frequency of every 3-6 months. They will review progress with recruitment and data collection.

This study will be registered with clinical trial registries including ClinicalTrials.gov.

Archiving of data

During this study data will be stored in a protected, encrypted database on Royal Free Hospital computers in secure locations. This data will only be accessible by members of the study team. Following study completion this data will be retained in the secure database on the Royal Free system.

Patient and public involvement in this study

The idea for this study arose from an appreciation of the burden of acute respiratory illness within an apparently healthy HIV infected population using antiretroviral therapy. It is essential that this research answers questions that are of importance to the population studied and subsequent discussions with patients from the Royal Free HIV ambulatory care service have focussed on study design and planning. This has been with the aim of developing a methodology that is acceptable to patients and in line with what they feel would be important outputs from this study. I have therefore involved members of the HIV patient users group, who have expressed considerable interest and enthusiasm regarding the study and provided valuable feedback concerning recruitment and retention of subjects in this study. We see this as an on-going dialogue and will positively encourage feedback on methodology as the study progresses. Ms Sophie Strachan (Senior Peer Case Worker, Positively UK) has also been involved in study planning and has offered her support to the project. The study will be performed in line with INVOLVE principles. Information of relevance will be fed back to both individuals involved with the study and also other service users and the wider public.

Translation into clinical benefit.

This study will prospectively document for the first time the frequency of respiratory tract illness in a UK HIV infected population. The major research output of this study will be an improved understanding of the rates of respiratory tract infection and respiratory illness in general in HIV infected individuals, the majority of whom will be on successful antiretroviral therapy. The evidence suggests that we will find high rates of reported symptoms and a large associated healthcare utilisation.

We will be able to explore the relationship between respiratory illness and smoking and other cofactors, including other lifestyle factors. The data from this will enable us to formulate public health and policy measures intended to reduce the frequency of respiratory illness. These may include smoking cessation strategies tailored for an HIV infected population, greater promotion of immunisation against influenza and pneumococcus and more intensive treatment of acute respiratory illnesses.[52,53,54]

A better understanding of the burden of respiratory disease, and the pathogens responsible for respiratory tract infection, could allow available healthcare resources to be more efficiently utilised. In the longer term, if respiratory illnesses are a factor driving the development of chronic lung disease, we would hope that interventions to reduce their frequency would lead to a reduction in these conditions as well.

Confidentiality

Full patient confidentiality will be maintained throughout the study, with access to samples and data restricted to the study investigators and their deputies.

Overview of study

Recruitment of participants and collection of baseline data. 140 HIV infected individuals and 70 HIV uninfected individuals.

↓

Participants record any symptoms of acute respiratory illness each week

↓

During times of acute respiratory illness participants reviewed by study team (and treated as clinically indicated).

Collection of sputum and nasopharyngeal swabs.

Completion of questionnaires to determine severity and of diary cards to determine duration of illness.

↓

Subjects routinely reviewed every 4 months. Questionnaires regarding symptoms and relevant exposures (eg smoking) repeated.

Spirometry repeated annually

↓

After 1 year study complete.

Analysis of results.

↓

Participant invited to enter longer-term follow-up cohort

Project Personnel

Project Lead:

Dr. Marc Lipman
Senior Clinical Lecturer
Consultant Respiratory/HIV Medicine,
Royal Free London NHS Foundation Trust,
The Grove Respiratory Centre
Pond Street
London NW3 2PF
marclipman@.nhs.net

Primary Researcher:

Dr James Brown
Respiratory Registrar
Department of Respiratory Medicine,
Royal Free London NHS Foundation Trust,
Pond Street
London NW3 2PF
james.brown13@nhs.net

Project supervisors:

Marc Lipman, Royal Free Hospital and UCL.

(details as above)

Professor Margaret Johnson
Consultant Respiratory/HIV Medicine
Royal Free London NHS Foundation Trust,
Rowland Hill Street
London NW3 2PF

Professor Ibrahim Abubakar
Professor in Infectious Disease Epidemiology
4th Floor, Mortimer Market Centre
Off Capper Street
London
WC1E 6JB

Collaborators:

Professor Steve Morris
Chair of Health Economics
Department of Applied Health Research
1-19 Torrington Place
London WC1E 7HB
steve.morris@ucl.ac.uk

Professor Tim McHugh
Professor of Medical Microbiology
Research Department of Infection
Centre for Clinical Microbiology,
Royal Free Campus
Rowland Hill Street
London NW4 2QG
t.mchugh@ucl.ac.uk

Dr Alison Rodger
Senior Lecturer and Honorary Consultant in Infectious Diseases,
Infection and Population Health
Royal Free London NHS Foundation Trust,
Pond Street
London NW3 2PF

alison.rodger@ucl.ac.uk

Dr Colette Smith
Lecturer in Biostatistics
Infection and Population Health
Royal Free London NHS Foundation Trust,
Pond Street
London NW3 2PF

c.smith@ucl.ac.uk

Dr John Hurst,
Reader in Respiratory Medicine,
Consultant Respiratory Physician,
University College London,
Royal Free London NHS Foundation Trust,
Pond Street
London NW3 2PF

j.hurst@ucl.ac.uk

Dr Daniel Webster
Consultant Virologist
Royal Free London NHS Foundation Trust,

Rowland Hill Street
London NW3 2PF

danielwebster@nhs.net

References

[1]Wada N, Jacobson LP, Cohen M, French A, Phair J, Muñoz A. **Cause-specific life expectancies after 35 years of age for human immunodeficiency syndrome-infected and human immunodeficiency syndrome-negative individuals followed simultaneously in long-term cohort studies, 1984-2008.** Am J Epidemiol. 2013 Jan 15;177(2):116-25.

[2]Nakagawa F, May M, Phillips A. **Life expectancy living with HIV: recent estimates and future implications.** CurrOpin Infect Dis. 2013 Feb;26(1):17-25.

[3]Morris A, Crothers K, Beck JM, Huang L; American Thoracic Society Committee on HIV Pulmonary Disease.**An official ATS workshop report: Emerging issues and current controversies in HIV-associated pulmonary diseases**.Proc Am Thorac Soc. 2011 Mar;8(1):17-26.

[4] Gingo MR, George MP, Kessinger CJ, Lucht L, Rissler B, Weinman R, Slivka WA, McMahon DK, Wenzel SE, Sciurba FC, Morris A. **Pulmonary function abnormalities in HIV-infected** patients during the current antiretroviral therapy era. Am J Respir Crit Care Med. 2010 Sep 15;182(6):790-6

[5] Crothers K, Butt AA, Gibert CL, Rodriguez-Barradas MC, Crystal S, Justice AC: Increased COPD among HIV-positive compared to HIV-negative veterans. Chest 2006, 130:1326-1333.

[6] Wallace JM, Hansen NI, Lavange L, Glassroth J, Browdy BL, Rosen MJ, Kvale PA, Mangura BT, Reichman LB, Hopewell PC. **Respiratory disease trends in the Pulmonary Complications of HIV Infection Study cohort.** Pulmonary Complications of HIV Infection Study Group. Am J Respir Crit Care Med. 1997 Jan;155(1):72-80.

[7]Matthew R. Gingo, G. K. Balasubramani, Lawrence Kingsley, *et al***The Impact of HAART on the Respiratory Complications of HIV Infection: Longitudinal Trends in the MACS and WIHS Cohorts** PLoS One. 2013; 8(3): e58812.

[8]Crothers K, Huang L, Goulet JL, *et al* **HIV infection and risk for incident pulmonary diseases in the combination antiretroviral therapy era.** Am J Respir Crit Care Med. 2011 Feb 1;183(3):388-95.

[9]Klein MB, Yang H, DelBalso L, Carbonneau J, Frost E, Boivin G. **Viral pathogens including human metapneumovirus are the primary cause of febrile respiratory illness in HIV-infected adults receiving antiretroviral therapy.** J Infect Dis. 2010 Jan 15;201(2):297-301.

[10]Morris A, Crothers K, Beck JM, Huang L; American Thoracic Society Committee on HIV Pulmonary Disease.**An official ATS workshop report: Emerging issues and current controversies in HIV-associated pulmonary diseases**.Proc Am Thorac Soc. 2011 Mar;8(1):17-26.

[11] Drummond MB[1], Merlo CA, Astemborski J, Kalmin MM, Kisalu A, Mcdyer JF, Mehta SH, Brown RH, Wise RA, Kirk GD. **The effect of HIV infection on longitudinal lung function decline among IDUs: a prospective cohort.** AIDS. 2013 May 15;27(8):1303-11.

[12] Drummond MB, Kirk GD, Ricketts EP, McCormack MC, Hague JC, McDyer JF, Mehta SH, Engels EA, Wise RA, Merlo CA: **Cross sectional analysis of respiratory symptoms in an injection drug user cohort: the impact of obstructive lung disease and HIV**. BMC Pulm Med 2010, 10:27.

[13]Madeddu G, Fois AG, Calia GM, Babudieri S, Soddu V, Becciu F, Fiori ML, Spada V, Lovigu C, Mannazzu M, Caddeo A, Piras B, Pirina P, Mura MS. **Chronic obstructive pulmonary disease: an emerging comorbidity in HIV-infected patients in the HAART era?**Infection. 2013 Apr;41(2):347-53.

[14] Crothers K, McGinnis K, Kleerup E, Wongtrakool C, Hoo GS, Kim J, Sharafkhaneh A, Huang L, Luo Z, Thompson B, Diaz P, Kirk GD, Rom W, Detels R, Kingsley L, Morris A. **HIV infection** is **associated with reduced pulmonary diffusing capacity**. J Acquir Immune Defic Syndr. 2013 Nov 1;64(3):271-8

[15] Campo M, Oursler KK, Huang L, Goetz M, Rimland D, Hoo GS, Brown S, Rodriguez-Barradas M, Au D, Akgün KM, Shahrir S, Crothers K. **Association of Chronic Cough and Pulmonary Function with 6-Minute Walk Test Performance in HIV Infection.** J Acquir Immune Defic Syndr. 2013 Dec 16.

[16] Kendall CE, Wong J, Taljaard M, Glazier RH, Hogg W, Younger J, Manuel DG. **A cross-sectional, population-based study measuring comorbidity among people living with HIV in Ontario.** BMC Public Health. 2014 Feb 13;14:161.

[17] Miners AH[1], Sabin CA, Mocroft A, Youle M, Fisher M, Johnson M. **Health-related quality of life in individuals infected with HIV in the era of HAART.** HIV Clin Trials. 2001 Nov-Dec;2(6):484-92.

[18] Protopopescu C[1], Marcellin F, Spire B, Préau M, Verdon R, Peyramond D, Raffi F, Chêne G, Leport C, Carrieri MP. **Health-related quality of life in HIV-1-infected patients on HAART: a five-years longitudinal analysis accounting for dropout in the APROCO-COPILOTE cohort (ANRS CO-8).** Qual Life Res. 2007 May;16(4):577-91.

[19]Wu AW, Hanson KA, Harding G. *et al* **Responsiveness of the MOS-HIV and EQ-5D in HIV-infected adults receiving antiretroviral therapies**.Health and Quality of Live Outcomes 2013, **11**:42.

[20] Clayson DJ, Wild DJ, Quarterman P, Duprat-Lomon I, Kubin M, Coons SJ. **A comparative review of health-related quality-of-life measures for use in HIV/AIDS clinical trials.** Pharmacoeconomics. 2006;24(8):751-65.

[21]Jones PW, Quirk FH, Baveystock CM. **The St George's Respiratory Questionnaire.** Resp Med1991;**85**(SuppB33-27) 25-31.

[22] Jin Y, Liu Z, Wang X, Liu H, Ding G, Su Y, Zhu L, Wang N. **A systematic review of cohort studies of the quality of life in HIV/AIDS patients after antiretroviral therapy.** Int J STD AIDS. 2014 Mar 5.

[23] Mathews WC, May S. **EuroQol (EQ-5D) measure of quality of life predicts mortality, emergency department utilization, and hospital discharge rates in HIV-infected adults under care.** EuroQol 2007 Jan 25;5:5.

[24] Jones PW, Quirk FH, Baveystock CM, Littlejohns **A self-complete measure of health status for chronic airflow limitation. The St. George's Respiratory Questionnaire.** Am Rev Respir Dis. 1992 Jun;145(6):1321-7.

[25] **Campo** M, Oursler KK, Huang L, Goetz MB, Rimland D, Hoo GS, Brown ST, Rodriguez-Barradas MC, Au D, Akgün KM, Shahrir S, Crothers K. **Association of Chronic Cough and Pulmonary Function With 6-Minute Walk Test Performance in HIV Infection.** J Acquir Immune Defic Syndr. 2014 Apr 15;65(5):557-63.

[26] Hirani A, Cavallazzi R, Vasu T, Pachinburavan M, Kraft WK, Leiby B, Short W, Desimone J, Squires KE, Weibel S, Kane GC. **Prevalence of obstructive lung disease in HIV population: a cross sectional study.** Respir Med. 2011 Nov;105(11):1655-65.

[27] Drummond MB[1], Kirk GD, McCormack MC, Marshall MM, Ricketts EP, Mehta SH, Wise RA, Merlo CA **HIV and COPD: impact of risk behaviors and diseases on quality of life.** Qual Life Res. 2010 Nov;19(9):1295-302.

[28]Ashworth M, Charlton J, Ballard K, Latinovic R, Gulliford M**. Variations in antibiotic prescribing and consultation rates for acute respiratory infection in UK general practices 1995-2000**.Br J Gen Pract. 2005 Aug;55(517):603-8

[29]**Respiratory Tract Infections - Antibiotic Prescribing: Prescribing of Antibiotics for Self-Limiting Respiratory Tract Infections in Adults and Children in Primary Care.**Centre for Clinical Practice at **NICE** (UK). London: National Institute for Health and Clinical Excellence (UK); 2008 Jul.

[30]Macfarlane J, Holmes W, Gard P, Macfarlane R, Rose D, Weston V, Leinonen M, Saikku P, Myint S. **Prospective study of the incidence, aetiology and outcome of adult lower respiratory tract illness in the community**.Thorax. 2001 Feb;56(2):109-14.

[31] Hayward AC[1], Fragaszy EB[2], Bermingham A **Comparative community burden and severity of seasonal and pandemic influenza: results of the Flu Watch cohort study.** Lancet Respir Med. 2014 Mar 17 (epub ahead of print).

[32] Bonafede MM[1], Suaya JA, Wilson KL, Mannino DM, Polsky D. **Incidence and cost of CAP in a large working-age population.** Am J Manag Care. 2012 Jul;18(7):380-7.

[33]http://handbook.cochrane.org/

[34]http://www.prisma-statement.org/

[35]Wu AW, Hanson KA, Harding G. *et al* **Responsiveness of the MOS-HIV and EQ-5D in HIV-infected adults receiving antiretroviral therapies**.Health and Quality of Live Outcomes 2013, **11**:42.

[36]Jones PW, Quirk FH, Baveystock CM. **The St George's Respiratory Questionnaire.**Resp Med 1991;**85**(SuppB33-27) 25-31.

[37] Mackay AJ, Donaldson GC, Patel ARC **Detection and severity grading of COPD exacerbations using the exacerbations of chronic pulmonary disease tool (EXACT)** Eur Respir J 2014:43 735-744

[38] Bjermer L[1], Alving K **Current evidence and future research needs for FeNO measurement in respiratory diseases.** Respir Med. 2014 Feb 15. [Epub ahead of print]

[39] Patel AR, Kowlessar BS, Donaldson GC, Mackay AJ, Singh R, George SN, Garcha DS, Wedzicha JA, Hurst JR. **Cardiovascular risk, myocardial injury, and exacerbations of chronic obstructive pulmonary disease.** Am J Respir Crit Care Med. 2013 Nov 1;188(9):1091-9.

[40] Iadarola P, Luisetti M. **The role of desmosines as biomarkers for chronic obstructive pulmonary disease.** Expert Rev Respir Med. 2013 Apr;7(2):137-44

[41]Molyneaux PL, Mallia P, Cox MJ, Footitt J, *et al* .**Outgrowth of the bacterial airway microbiome after rhinovirus exacerbation of chronic obstructive pulmonary disease** .Am J RespirCrit Care Med. 2013 Nov 15;188(10):1224-31.

[42]Burge S, Wedzicha JA. **COPD exacerbations: definitions and classifications**. EurRespir J Suppl. 2003 Jun;41:46s-53s

[43]Lim WS, Baudouin SV, George RC, Hill AT, Jamieson C, Le Jeune I, Macfarlane JT, Read RC, Roberts HJ, Levy ML, Wani M, Woodhead MA; **Pneumonia Guidelines Committee of the BT SStandards of Care Committee.** BTS guidelines for the management of community acquired pneumonia in adults: update 2009.Thorax. 2009 Oct;64Suppl 3:iii1-55.

[44] Monto AS, Napier JA, Metzner HL. **The Tecumseh study of respiratory illness. I. Plan of study and observations on syndromes of acute respiratory disease.**Am J Epidemiol. 1971 Sep;94(3):269-79.

[45] WHO Global Epidemiological Surveillance Standards for Influenza 2012

[46]Hope-Simpson RE, Miller DL. **The definition of acute respiratory illnesses in general practice.** Postgrad Med J. 1973 Nov;49(577):763-70.

[47] Macfarlane J, Holmes W, Gard P, Macfarlane R, Rose D, Weston V, Leinonen M, Saikku P, Myint S**. Prospective study of the incidence, aetiology and outcome of adult lower respiratory tract illness in the community.** Thorax. 2001 Feb;56(2):109-14.

[48] Bryony W. Soltis, John W. Sanders, Shannon D. Putnam, David R. Tribble, Mark S. Riddle **Self-Reported Incidence and Morbidity of Acute Respiratory Illness among Deployed U.S. Military in Iraq and Afghanistan** PLoS One. 2009; 4(7): e6177

[49] Bayer C, Remschmidt C, an der Heiden M, Tolksdorf K, Herzhoff M, Kaersten S, Buda S, Haas W, Buchholz U. **Internet-based syndromic monitoring of acute respiratory illness in the general population of Germany, weeks 35/2011 to 34/2012.** Euro Surveill. 2014 Jan 30;19(4). pii: 20684.

[50] Ortiz JR, Sotomayor V, Uez OC, et al. **Strategy to enhance influenza surveillance worldwide**. Emerg Infect Dis 2009;15:1271–8

[51] Lim WS, Baudouin SV, George RC, Hill AT, Jamieson C, Le Jeune I, Macfarlane JT, Read RC, Roberts HJ, Levy ML, Wani M, Woodhead MA; Pneumonia Guidelines Committee of the BTS Standards of Care Committee **BTS guidelines for the management of community acquired pneumonia in adults: update 2009.** Thorax. 2009 Oct;64 Suppl 3:iii1-55. doi: 10.1136/thx.2009.121434.

[52]Keri N. Althoff, Kathryn Anastos, Kenrad E *et al*. **Nelson Predictors of reported influenza vaccination in HIV-infected women in the United States, 2006-07 and 2007-08 seasons**, Prev Med. 2010 May–Jun; 50(5-6): 223–229.

[53]De P, Farley A, Lindson N, AveyardP.**Systematic review and meta-analysis: influence of smoking cessation on incidence of pneumonia in HIV.** BMC Med. 2013 Jan 22;11:15.

[54]Smith KJ, Nowalk MP, Raymund M, Zimmerman RK.**Cost-effectiveness of pneumococcal conjugate vaccination in immunocompromised adults.** Vaccine. 2013 Aug 20;31(37):3950-6.
