# Supplementary material for: The effect of HIV status on the frequency and severity of acute respiratory illness
Source: PLoS One. 2020 May 29;15(5):e0232977. doi: 10.1371/journal.pone.0232977 (PMC7259631; doi:10.1371/journal.pone.0232977)
Supplement: S2 Data — (PDF) [file pone.0232977.s002.pdf]

Royal Free London NHS Foundation Trust NHS

ACUTE RESPIRATORY ILLNESS SYMPTOM DIARY

Many thanks for participating in this study.

You are completing this diary because you have told us that you currently have new respiratory symptoms (such as a cough, blocked nose or sore throat).

This diary will ask you to assess how severe these symptoms are each day, for up to 14 days, or until you feel back to normal.

Please complete this diary at the end of each day.

Please also contact the study team, to arrange a time to be reviewed and collect samples to see if we can detect any viruses or bacteria that might be causing your illness.

DAY 1

Please tick which day:

Monday	Tuesday	Wednesday	Thursday	Friday	Saturday	Sunday
○	○	○	○	○	○	○

For each of the symptoms listed, please indicate how severe it is today by ticking one of the boxes in each row:

	NO PROBLEM	MILD		MODERATE		SEVERE PROBLEM	
**SYMPTOM**	**0**	**1**	**2**	**3**	**4**	**5**	**6**
Sore throat							
Blocked nose							
Fever or chills							
Cough							
Producing more phlegm (sputum) than normal							
Shortness of breath							
Disturbed sleep							
Is your illness interfering with your normal activity?							
Overall, how unwell do you feel?							

Have you seen a doctor, nurse or pharmacist about this illness? (please tick)

No ◯ Yes, a pharmacist ◯ Yes a nurse in my GP practice ◯ Yes, in a hospital clinic ◯

Yes a doctor in my GP practice ◯ Yes, in a hospital accident and emergency department ◯

Are you taking any treatment for this illness? (please tick)

No ◯ Yes, over the counter from a pharmacy ◯ Yes, I am taking antibiotics ◯

Yes, other treatment.
Please state

DAY 2

Please tick which day:

Monday	Tuesday	Wednesday	Thursday	Friday	Saturday	Sunday
○	○	○	○	○	○	○

For each of the symptoms listed, please indicate how severe it is today by ticking one of the boxes in each row:

	NO PROBLEM	MILD		MODERATE		SEVERE PROBLEM	
**SYMPTOM**	**0**	**1**	**2**	**3**	**4**	**5**	**6**
Sore throat							
Blocked nose							
Fever or chills							
Cough							
Producing more phlegm (sputum) than normal							
Shortness of breath							
Disturbed sleep							
Is your illness interfering with your normal activity?							
Overall, how unwell do you feel?							

Have you seen a doctor, nurse or pharmacist about this illness? (please tick)

No ○ Yes, a pharmacist ○ Yes a nurse in my GP practice ○ Yes, in a hospital clinic ○

Yes a doctor in my GP practice ○ Yes, in a hospital accident and emergency department ○

Are you taking any treatment for this illness? (please tick)

No ○ Yes, over the counter from a pharmacy ○ Yes, I am taking antibiotics ○

Yes, other treatment.
Please state

DAY 3

Please tick which day:

Monday	Tuesday	Wednesday	Thursday	Friday	Saturday	Sunday
○	○	○	○	○	○	○

For each of the symptoms listed, please indicate how severe it is today by ticking one of the boxes in each row:

	NO PROBLEM	MILD		MODERATE		SEVERE PROBLEM	
**SYMPTOM**	**0**	**1**	**2**	**3**	**4**	**5**	**6**
Sore throat							
Blocked nose							
Fever or chills							
Cough							
Producing more phlegm (sputum) than normal							
Shortness of breath							
Disturbed sleep							
Is your illness interfering with your normal activity?							
Overall, how unwell do you feel?							

Have you seen a doctor, nurse or pharmacist about this illness? (please tick)

No ◯ Yes, a pharmacist ◯ Yes a nurse in my GP practice ◯ Yes, in a hospital clinic ◯

Yes a doctor in my GP practice ◯ Yes, in a hospital accident and emergency department ◯

Are you taking any treatment for this illness? (please tick)

No ◯ Yes, over the counter from a pharmacy ◯ Yes, I am taking antibiotics ◯

Yes, other treatment.
Please state

DAY 4

Please tick which day:

Monday	Tuesday	Wednesday	Thursday	Friday	Saturday	Sunday
○	○	○	○	○	○	○

For each of the symptoms listed, please indicate how severe it is today by ticking one of the boxes in each row:

	NO PROBLEM	MILD		MODERATE		SEVERE PROBLEM	
**SYMPTOM**	**0**	**1**	**2**	**3**	**4**	**5**	**6**
Sore throat							
Blocked nose							
Fever or chills							
Cough							
Producing more phlegm (sputum) than normal							
Shortness of breath							
Disturbed sleep							
Is your illness interfering with your normal activity?							
Overall, how unwell do you feel?							

Have you seen a doctor, nurse or pharmacist about this illness? (please tick)

No ◯ Yes, a pharmacist ◯ Yes a nurse in my GP practice ◯ Yes, in a hospital clinic ◯

Yes a doctor in my GP practice ◯ Yes, in a hospital accident and emergency department ◯

Are you taking any treatment for this illness? (please tick)

No ◯ Yes, over the counter from a pharmacy ◯ Yes, I am taking antibiotics ◯

Yes, other treatment.
Please state

DAY 5

Please tick which day:

Monday	Tuesday	Wednesday	Thursday	Friday	Saturday	Sunday
○	○	○	○	○	○	○

For each of the symptoms listed, please indicate how severe it is today by ticking one of the boxes in each row:

	NO PROBLEM	MILD		MODERATE		SEVERE PROBLEM	
**SYMPTOM**	**0**	**1**	**2**	**3**	**4**	**5**	**6**
Sore throat							
Blocked nose							
Fever or chills							
Cough							
Producing more phlegm (sputum) than normal							
Shortness of breath							
Disturbed sleep							
Is your illness interfering with your normal activity?							
Overall, how unwell do you feel?							

Have you seen a doctor, nurse or pharmacist about this illness? (please tick)

No ◯ Yes, a pharmacist ◯ Yes a nurse in my GP practice ◯ Yes, in a hospital clinic ◯

Yes a doctor in my GP practice ◯ Yes, in a hospital accident and emergency department ◯

Are you taking any treatment for this illness? (please tick)

No ◯ Yes, over the counter from a pharmacy ◯ Yes, I am taking antibiotics ◯

Yes, other treatment.
Please state

DAY 6

Please tick which day:

Monday	Tuesday	Wednesday	Thursday	Friday	Saturday	Sunday
○	○	○	○	○	○	○

For each of the symptoms listed, please indicate how severe it is today by ticking one of the boxes in each row:

	NO PROBLEM	MILD		MODERATE		SEVERE PROBLEM	
SYMPTOM	0	1	2	3	4	5	6
Sore throat							
Blocked nose							
Fever or chills							
Cough							
Producing more phlegm (sputum) than normal							
Shortness of breath							
Disturbed sleep							
Is your illness interfering with your normal activity?							
Overall, how unwell do you feel?							

Have you seen a doctor, nurse or pharmacist about this illness? (please tick)

No ○ Yes, a pharmacist ○ Yes a nurse in my GP practice ○ Yes, in a hospital clinic ○

Yes a doctor in my GP practice ○ Yes, in a hospital accident and emergency department ○

Are you taking any treatment for this illness? (please tick)

No ○ Yes, over the counter from a pharmacy ○ Yes, I am taking antibiotics ○

Yes, other treatment.
Please state

DAY 7

Please tick which day:

Monday	Tuesday	Wednesday	Thursday	Friday	Saturday	Sunday
○	○	○	○	○	○	○

For each of the symptoms listed, please indicate how severe it is today by ticking one of the boxes in each row:

	NO PROBLEM	MILD		MODERATE		SEVERE PROBLEM	
**SYMPTOM**	**0**	**1**	**2**	**3**	**4**	**5**	**6**
Sore throat							
Blocked nose							
Fever or chills							
Cough							
Producing more phlegm (sputum) than normal							
Shortness of breath							
Disturbed sleep							
Is your illness interfering with your normal activity?							
Overall, how unwell do you feel?							

Have you seen a doctor, nurse or pharmacist about this illness? (please tick)

No ◯ Yes, a pharmacist ◯ Yes a nurse in my GP practice ◯ Yes, in a hospital clinic ◯

Yes a doctor in my GP practice ◯ Yes, in a hospital accident and emergency department ◯

Are you taking any treatment for this illness? (please tick)

No ◯ Yes, over the counter from a pharmacy ◯ Yes, I am taking antibiotics ◯

Yes, other treatment.
Please state

DAY 8

Please tick which day:

Monday	Tuesday	Wednesday	Thursday	Friday	Saturday	Sunday
○	○	○	○	○	○	○

For each of the symptoms listed, please indicate how severe it is today by ticking one of the boxes in each row:

	NO PROBLEM	MILD		MODERATE		SEVERE PROBLEM	
**SYMPTOM**	**0**	**1**	**2**	**3**	**4**	**5**	**6**
Sore throat							
Blocked nose							
Fever or chills							
Cough							
Producing more phlegm (sputum) than normal							
Shortness of breath							
Disturbed sleep							
Is your illness interfering with your normal activity?							
Overall, how unwell do you feel?							

Have you seen a doctor, nurse or pharmacist about this illness? (please tick)

- No
- Yes, a pharmacist
- Yes a nurse in my GP practice
- Yes, in a hospital clinic
- Yes a doctor in my GP practice
- Yes, in a hospital accident and emergency department

Are you taking any treatment for this illness? (please tick)

- No
- Yes, over the counter from a pharmacy
- Yes, I am taking antibiotics

Yes, other treatment.
Please state

DAY 9

Please tick which day:

Monday	Tuesday	Wednesday	Thursday	Friday	Saturday	Sunday
○	○	○	○	○	○	○

For each of the symptoms listed, please indicate how severe it is today by ticking one of the boxes in each row:

	NO PROBLEM	MILD		MODERATE		SEVERE PROBLEM	
SYMPTOM	0	1	2	3	4	5	6
Sore throat							
Blocked nose							
Fever or chills							
Cough							
Producing more phlegm (sputum) than normal							
Shortness of breath							
Disturbed sleep							
Is your illness interfering with your normal activity?							
Overall, how unwell do you feel?							

Have you seen a doctor, nurse or pharmacist about this illness? (please tick)

No ◯ Yes, a pharmacist ◯ Yes a nurse in my GP practice ◯ Yes, in a hospital clinic ◯

Yes a doctor in my GP practice ◯ Yes, in a hospital accident and emergency department ◯

Are you taking any treatment for this illness? (please tick)

No ◯ Yes, over the counter from a pharmacy ◯ Yes, I am taking antibiotics ◯

Yes, other treatment.
Please state

DAY 10

Please tick which day:

Monday	Tuesday	Wednesday	Thursday	Friday	Saturday	Sunday
○	○	○	○	○	○	○

For each of the symptoms listed, please indicate how severe it is today by ticking one of the boxes in each row:

	NO PROBLEM	MILD		MODERATE		SEVERE PROBLEM	
**SYMPTOM**	**0**	**1**	**2**	**3**	**4**	**5**	**6**
Sore throat							
Blocked nose							
Fever or chills							
Cough							
Producing more phlegm (sputum) than normal							
Shortness of breath							
Disturbed sleep							
Is your illness interfering with your normal activity?							
Overall, how unwell do you feel?							

Have you seen a doctor, nurse or pharmacist about this illness? (please tick)

No ◯ Yes, a pharmacist ◯ Yes a nurse in my GP practice ◯ Yes, in a hospital clinic ◯

Yes a doctor in my GP practice ◯ Yes, in a hospital accident and emergency department ◯

Are you taking any treatment for this illness? (please tick)

No ◯ Yes, over the counter from a pharmacy ◯ Yes, I am taking antibiotics ◯

Yes, other treatment.
Please state

DAY 11

Please tick which day:

Monday ◯ Tuesday ◯ Wednesday ◯ Thursday ◯ Friday ◯ Saturday ◯ Sunday ◯

For each of the symptoms listed, please indicate how severe it is today by ticking one of the boxes in each row:

	NO PROBLEM	MILD		MODERATE		SEVERE PROBLEM	
SYMPTOM	0	1	2	3	4	5	6
Sore throat							
Blocked nose							
Fever or chills							
Cough							
Producing more phlegm (sputum) than normal							
Shortness of breath							
Disturbed sleep							
Is your illness interfering with your normal activity?							
Overall, how unwell do you feel?							

Have you seen a doctor, nurse or pharmacist about this illness? (please tick)

No ◯ Yes, a pharmacist ◯ Yes a nurse in my GP practice ◯ Yes, in a hospital clinic ◯

Yes a doctor in my GP practice ◯ Yes, in a hospital accident and emergency department ◯

Are you taking any treatment for this illness? (please tick)

No ◯ Yes, over the counter from a pharmacy ◯ Yes, I am taking antibiotics ◯

Yes, other treatment.
Please state

DAY 12

Please tick which day:

Monday ○ Tuesday ○ Wednesday ○ Thursday ○ Friday ○ Saturday ○ Sunday ○

For each of the symptoms listed, please indicate how severe it is today by ticking one of the boxes in each row:

	NO PROBLEM	MILD		MODERATE		SEVERE PROBLEM	
SYMPTOM	0	1	2	3	4	5	6
Sore throat							
Blocked nose							
Fever or chills							
Cough							
Producing more phlegm (sputum) than normal							
Shortness of breath							
Disturbed sleep							
Is your illness interfering with your normal activity?							
Overall, how unwell do you feel?							

Have you seen a doctor, nurse or pharmacist about this illness? (please tick)

- No
- Yes, a pharmacist
- Yes a nurse in my GP practice
- Yes, in a hospital clinic
- Yes a doctor in my GP practice
- Yes, in a hospital accident and emergency department

Are you taking any treatment for this illness? (please tick)

- No
- Yes, over the counter from a pharmacy
- Yes, I am taking antibiotics

Yes, other treatment.
Please state

DAY 13

Please tick which day:

Monday	Tuesday	Wednesday	Thursday	Friday	Saturday	Sunday
○	○	○	○	○	○	○

For each of the symptoms listed, please indicate how severe it is today by ticking one of the boxes in each row:

	NO PROBLEM	MILD		MODERATE		SEVERE PROBLEM	
**SYMPTOM**	**0**	**1**	**2**	**3**	**4**	**5**	**6**
Sore throat							
Blocked nose							
Fever or chills							
Cough							
Producing more phlegm (sputum) than normal							
Shortness of breath							
Disturbed sleep							
Is your illness interfering with your normal activity?							
Overall, how unwell do you feel?							

Have you seen a doctor, nurse or pharmacist about this illness? (please tick)

No ◯ Yes, a pharmacist ◯ Yes a nurse in my GP practice ◯ Yes, in a hospital clinic ◯

Yes a doctor in my GP practice ◯ Yes, in a hospital accident and emergency department ◯

Are you taking any treatment for this illness? (please tick)

No ◯ Yes, over the counter from a pharmacy ◯ Yes, I am taking antibiotics ◯

Yes, other treatment.
Please state

DAY 14

Please tick which day:

Monday	Tuesday	Wednesday	Thursday	Friday	Saturday	Sunday

For each of the symptoms listed, please indicate how severe it is today by ticking one of the boxes in each row:

	NO PROBLEM	MILD		MODERATE		SEVERE PROBLEM	
**SYMPTOM**	**0**	**1**	**2**	**3**	**4**	**5**	**6**
Sore throat							
Blocked nose							
Fever or chills							
Cough							
Producing more phlegm (sputum) than normal							
Shortness of breath							
Disturbed sleep							
Is your illness interfering with your normal activity?							
Overall, how unwell do you feel?							

Have you seen a doctor, nurse or pharmacist about this illness? (please tick)

No ○ Yes, a pharmacist ○ Yes a nurse in my GP practice ○ Yes, in a hospital clinic ○

Yes a doctor in my GP practice ○ Yes, in a hospital accident and emergency department ○

Are you taking any treatment for this illness? (please tick)

No ○ Yes, over the counter from a pharmacy ○ Yes, I am taking antibiotics ○

Yes, other treatment.
Please state

Many thanks for completing this diary.

If you have not already made arrangements to return this to the study team, please contact us by email at: **rf.acuterespiratory@nhs.net** or phone us on: **07468 934026** (you can call any time, please leave a message and we'll get back to you).

The acute respiratory illness project is funded by the National Institute for Health Research's Doctoral Research Fellowship Programme and the British HIV Association Research Award.

British HIV Association BHIVA

NHS National Institute for Health Research
