# Supplementary material for: The effect of HIV status on the frequency and severity of acute respiratory illness
Source: PLoS One. 2020 May 29;15(5):e0232977. doi: 10.1371/journal.pone.0232977 (PMC7259631; doi:10.1371/journal.pone.0232977)
Supplement: S4 Data — (PDF) [file pone.0232977.s004.pdf]

Respiratory Health and rates of acute respiratory illness questionnaire

We would very much appreciate your help with this study.

The following questionnaire has been designed to explore people's experience of respiratory illness and factors that might influence this. Please tick the boxes where appropriate. There are no right or wrong answers so please just select the answer that best suits you. We have also left some space for additional comments.

At the end, there are some quality of life questions about how you feel today. After that, we would like to check your blood pressure and then breathing with a machine called a spirometer, which works out how much air you can blow out of your lungs and is a measure of lung function. The questionnaire should not take more than 10 minutes to complete, then about 10 minutes to do the spirometry.

We are asking these questions to try and get a better understanding of the rates of respiratory symptoms and what influences these. We would also like to link this information to medications, blood test results (such as viral load and CD4 count) and previous illnesses from your medical records.

Many thanks for taking the time to help us with our study.

Your responses will be treated in complete confidence. Results will be stored in anonymised form.

Date of questionnaire:

Part A. About you:

Name: ……………………………………………………….

Hospital number (if known) …………………………….

Gender: Male □ Female □ (tick box)

Date of birth (dd/mm/yy) …………………………………….

Please choose the one which best describes you: (tick box)

Asian – Indian/ Pakistani/ Bangladeshi □

Black African □

Black Caribbean □

Black other □

Mixed white and black □

Mixed other □

White British □

White Irish □

White other □

Other (please state) : ………………………

Do you consider yourself:

Bisexual ☐

Heterosexual ☐

Homosexual ☐

Other (please state) : ………………………

What is your occupation?

How many people live in the same house or flat as you?

Are there any children under the age of 2 living in the same house or flat as you?

YES ☐ NO ☐

Do you have any children between the ages of 2 and 16 living in the same house or flat as you?

YES ☐ NO ☐

Part B. Your respiratory health and fitness:

1. How far can you walk? (related to activities - please tick the appropriate number)

☐ Not troubled by breathlessness except on strenuous exercise

☐ Short of breath when hurrying or walking up a slight hill

☐ Walk slower than contemporaries on level ground or have to stop for breath when walking at your own pace

☐ Stop for breath after walking about 100m or after a few minutes on level ground

☐ Too breathless to leave the house, or breathless when dressing/undressing

2. In the past 12 months, have you had any of the following illnesses? (Tick box)

Sinusitis	☐	Pneumonia	☐
Bronchitis	☐	Asthma	☐
Chest infection	☐	Pleurisy	☐

Cold or flu bad enough to make you miss work or normal activities ☐

Part C:

1. Which of the following best applies to you?

☐ a. I smoke cigarettes (including hand-rolled) every day

☐ b. I smoke cigarettes (including hand-rolled), but not every day

☐ c. I do not smoke cigarettes at all, but I do smoke tobacco of some kind (eg. pipe/cigar)

☐ d. I have stopped smoking completely in the last year

☐ e. I stopped smoking completely more than a year ago

☐ f. I have never been a smoker (i.e. smoked for a year or more)

If e – How old were you when you stopped smoking? (If you cannot remember the exact age, please provide an estimate) …….. years old

2. If you smoke, how many cigarettes **per week** do you usually smoke?……cigarettes

(how many are hand rolled ………………)

3. What age did you start smoking? ………years old

When you smoked the most, how many cigarettes did you smoke **a day**? ……….

4. Have you ever smoked cannabis? YES ☐ NO ☐

If yes: Once ever ☐

A few times ever ☐

Very infrequently ☐ (Number of times/year ....)

Regularly smoked for a period of time: ☐

Age started ...., no/week ...., age stopped .... (if applicable)

5. Have you ever used cocaine? YES □ NO □

If so: Smoked YES □ NO □

Inhaled/snorted (via nose) YES □ NO □

By mouth (rubbed on gums/swallowed) YES □ NO □

Injected YES □ NO □

Other…. YES □ NO □

If so, how regularly …………………………………..

6. Have you ever used heroin? YES □ NO □

If so: Smoked YES □ NO □

Injected YES □ NO □

Other drugs ……................... YES □ NO □

7. Were you exposed to cigarette smoke in the house as a child? YES □ NO □

8. Have you ever lived in a house that had an indoor stove that burned wood, coal or dung?

YES □ NO □

If so, when and for how long: ………………………

9. Do you know if you were born at term (40 weeks or 9 months) or if you were born early or late (if so by how much)?

UNSURE □ AT TERM □ EARLY □ ( ) LATE □ ( )

10. Did you have an influenza (flu) vaccine last winter (approximately between Oct and January)?

YES □ NO □

If so, where?

☐ At the Ian Charleson Centre

□ At my local GP surgery

□ At a pharmacy/supermarket

□ Other (please specify) ………………………………….

□ Don’t know

12. Have you ever had a pneumonia (pneumococcal) vaccine – otherwise called Pneumovax?

YES ☐ NO ☐

If so, where?

☐ At the Ian Charleson Centre

☐ At my local GP surgery

☐ At a pharmacy/supermarket

☐ Other (please specify) ………………………………….

☐ Don't know

In what part of the world were you born?

☐ UK

☐ Europe

☐ Asia

☐ North America

☐ South or Central America

☐ Africa

At present or in the past, have you ever had any of the following conditions?

☐ Pneumonia

☐ Pneumocystis (PCP) pneumonia

☐ Tuberculosis

☐ Asthma

☐ COPD (chronic obstructive pulmonary disease) or emphysema

SGRQ

Under each heading, please tick the ONE box that best describes your health TODAY

MOBILITY

I have no problems in walking about ❑
I have slight problems in walking about ❑
I have moderate problems in walking about ❑
I have severe problems in walking about ❑
I am unable to walk about ❑

SELF-CARE

I have no problems washing or dressing myself ❑
I have slight problems washing or dressing myself ❑
I have moderate problems washing or dressing myself ❑
I have severe problems washing or dressing myself ❑
I am unable to wash or dress myself ❑

USUAL ACTIVITIES *(e.g. work, study, housework, family or leisure activities)*

I have no problems doing my usual activities ❑
I have slight problems doing my usual activities ❑
I have moderate problems doing my usual activities ❑
I have severe problems doing my usual activities ❑
I am unable to do my usual activities ❑

PAIN / DISCOMFORT

I have no pain or discomfort ❑
I have slight pain or discomfort ❑
I have moderate pain or discomfort ❑
I have severe pain or discomfort ❑
I have extreme pain or discomfort ❑

ANXIETY / DEPRESSION

I am not anxious or depressed ❑
I am slightly anxious or depressed ❑
I am moderately anxious or depressed ❑
I am severely anxious or depressed ❑
I am extremely anxious or depressed ❑

UK (English) v.2 © 2009 EuroQol Group. EQ-5D™ is a trade mark of the EuroQol Group

- We would like to know how good or bad your health is TODAY.
- This scale is numbered from 0 to 100.
- 100 means the best health you can imagine.
  0 means the worst health you can imagine.
- Mark an X on the scale to indicate how your health is TODAY.
- Now, please write the number you marked on the scale in the box below.

YOUR HEALTH TODAY = ☐

The best health you can imagine

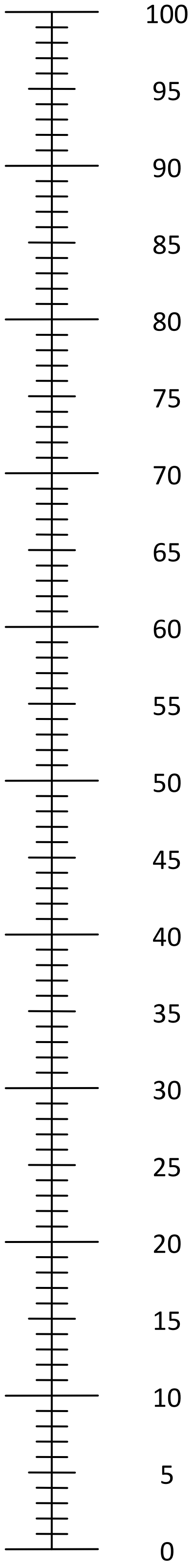

The worst health you can imagine

UK (English) v.2 © 2009 EuroQol Group. EQ-5D™ is a trade mark of the EuroQol Group

Many thanks for your involvement in this study.
