# Supplementary material for: The effect of HIV status on the frequency and severity of acute respiratory illness
Source: PLoS One. 2020 May 29;15(5):e0232977. doi: 10.1371/journal.pone.0232977 (PMC7259631; doi:10.1371/journal.pone.0232977)
Supplement: S5 Data — (PDF) [file pone.0232977.s005.pdf]

+Web Forms

Project Navigation
- NEXT PAGE
- PREVIOUS PAGE
- CANCEL
- CLEAR
- SUBMIT

Completion Errors
Page One (1)
Q1 New Onset
Minimum number of responses not found.

Royal Free London NHS
NHS Foundation Trust

Question 1
This week, have you had the *new onset* of *any* of the following symptoms:

Sore throat
Blocked or runny nose
Pain or pressure in the nose or face
Cough or increased production of phlegm (sputum)
Breathlessness or pain on breathing

Yes ☐ No ☐

+Web Forms

Project Navigation
- NEXT PAGE
- PREVIOUS PAGE
- CANCEL
- CLEAR
- SUBMIT

Completion Errors

Question 2
For each of the following problems, please indicate how much of a problem this is to you today. If you don't have one of these symptoms, please indicate "normal or no problem".

	Normal no problem	Very little problem	Slight problem	Moderately bad	Bad	Very bad	Bad as it could be
Sore throat	☐	☐	☐	☐	☐	☐	☐
Blocked nose or feeling of fullness or discomfort in the nose	☐	☐	☐	☐	☐	☐	☐
Fever or chills	☐	☐	☐	☐	☐	☐	☐
Cough	☐	☐	☐	☐	☐	☐	☐
Producing more phlegm (sputum) than normal	☐	☐	☐	☐	☐	☐	☐
Shortness of breath	☐	☐	☐	☐	☐	☐	☐
Disturbed sleep	☐	☐	☐	☐	☐	☐	☐
Is your illness interfering with your normal activity?	☐	☐	☐	☐	☐	☐	☐
Overall, how unwell do you feel?	☐	☐	☐	☐	☐	☐	☐

Question 3
Which day did you first become unwell?

Mon	Tues	Weds	Thurs	Fri	Sat	Sun
☐	☐	☐	☐	☐	☐	☐

+Web Forms

Question 4
Have you seen your GP or visited hospital regarding these symptoms?

☐ No

☐ Yes - Saw GP

☐ Yes - seen in hospital as outpatient

☐ Yes - admitted to hospital

Question 5
Are you taking any medications for your symptoms?

☐ I am not taking any medications for these symptoms

☐ Yes - I am taking pain killers, medications for fever (such as paracetamol or ibuprofen) or other medications that don't require a prescription

☐ Yes - antibiotics

+Web Forms

The following questions are about your general health today. For each one please tick the one box that best describes your health TODAY:

Question 6: Mobility

- ☐ I have no problems walking about
- ☐ I have slight problems walking about
- ☐ I have moderate problems walking about
- ☐ I have severe problems walking about
- ☐ I am unable to walk about

Question 7: Self-Care

- ☐ I have no problems washing or dressing myself
- ☐ I have slight problems washing or dressing myself
- ☐ I have moderate problems washing or dressing myself
- ☐ I have severe problems washing or dressing myself
- ☐ I am unable to wash or dress myself

Question 8: Usual Activities (e.g. work, study, house work, family or leisure activities)

- ☐ I have no problems doing my usual activities
- ☐ I have slight problems doing my usual activities
- ☐ I have moderate problems doing my usual activities
- ☐ I have severe problems doing my usual activities
- ☐ I am unable to do my usual activities

Question 9: Pain / Discomfort

☐ I have no pain or discomfort

☐ I have slight pain or discomfort

☐ I have moderate pain or discomfort

☐ I have severe pain or discomfort

☐ I have extreme pain or discomfort

Question 10: Anxiety / Depression

☐ I am not anxious or depressed

☐ I am slightly anxious or depressed

☐ I am moderately anxious or depressed

☐ I am severely anxious or depressed

☐ I am extremely anxious or depressed

Question 11

On a scale of 0 to 100, where 0 means WORST health you can imagine and 100 means BEST health you can imagine, how good or bad is your health today?

78

✖ 0 100

Click on **SUBMIT** to submit your data

Please could you contact the study team on telephone number 07468 934026 or email rf.acuterespiratory@nhs.net and we will arrange a convenient time to see you.

+Web Forms

The following questions are about your general health today. For each one please tick the one box that best describes your health TODAY:

Question 6: Mobility

☐ I have no problems walking about

☐ I have slight problems walking about

☐ I have moderate problems walking about

☐ I have severe problems walking about

☐ I am unable to walk about

Question 7: Self-Care

☐ I have no problems washing or dressing myself

☐ I have slight problems washing or dressing myself

☐ I have moderate problems washing or dressing myself

☐ I have severe problems washing or dressing myself

☐ I am unable to wash or dress myself
