# Supplementary material for: The effect of HIV status on the frequency and severity of acute respiratory illness
Source: PLoS One. 2020 May 29;15(5):e0232977. doi: 10.1371/journal.pone.0232977 (PMC7259631; doi:10.1371/journal.pone.0232977)
Supplement: Attachment — Submitted filename: review text.docx [file pone.0232977.s006.docx]

This is a very robust epidemiologic study looking at respiratory symptoms in a cohort of people living with HIV (PLwHIV) and relatively matched controls over a several year period of fairly robust regular communication. Respiratory illness was intentionally loosely defined to capture events that may result in communication with a healthcare professional or treatment. St. Georges Respiratory Questionaire (SGRQ) and the MRC dyspnea scale data was collected at baseline and during any acute illness. In addition an unweighted symptom score was collected daily during acute illness recovery. The PLwHIV were well controlled with all on ART and the majority with suppressed viral load. An attempt was made to identify the common viruses that were representative of the acute illnesses but this may be biased as more severe illness may result in less willingness to return for a swab. The control group is mostly age and gender matched with Ethnicity, sexuality, immunizations and respiratory medication use being different. The subjects had pre-bronchodilator spirometry and both restrictive and obstructive defects were more common in the PLwHIV. Associated with this both the SGRQ and MRC favored more symptoms in PLwHIV. The main outcome of the study, rate of acute illness was the same in both groups with most of the significant findings being related to the subjective measures of respiratory symptom severity. Consistent with would be predicted obstruction, prior smoking, and baseline dyspnea were all associated with greater illness frequency. Although the symptoms scores were higher and PLwHIV contacted medical providers more frequently use of non-prescription medications was equivalent. Overall 158 np swabs were evaluated and there was no difference in the rate of positive swabs between groups or in type of virus recovered. Overall the data is robust and discussed with appropriate notation of the limitations.

Suggestions:

1. The incidence of “restrictive” lung disease seems quite large in comparison to prior studies. This would be more appropriately called this PRISm (preserved ratio impaired spirometry) given the individuals only had pre-bronchodilator spirometry. This likely would also be consistent with a predilection for increased symptoms and likely an increased risk of future incident COPD. I do not think this has previously been described. This may be worth adding to the discussion given the findings of the Rotterdam Study would be consistent. From this standpoint it may be important to note who (trained respiratory therapist, research coordinator, nurse) performed the spirometry as an important caveat would be poor effort can give this sort of finding as well but would not explain the association only with PLwHIV.
2. Likey discussion should include the fact that the spirometry was all prebronchodilator and likely over represents the population as having COPD.
3. Inclusion of more information on the symptom score that was utilized at illness initiation and daily diaries would be beneficial. The presumption is that this symptom score was de novo invented and likely has not been confirmed to be of value as an unscaled entity in this disease process. Although this does not lessen the value of this information, knowing that the subscales of the SGRQ were all significantly more in PLwHIV suggests knowing more information about what makes up the Figure 1 results would help.
4. Figure 2 is not particularly well presented. The coloring is based on the percentage of each virus but likely would be improved if both “pie” graphs utilized the same color for same viruses. It is not clear to me if there is any difference in any measured virus as I suspect as the only notation in the text is that Rhinovirus is the most common in both groups and many viral swabs were negative. For instance it seems that influenza vaccination is more common in PLwHIV but yet isolating influenza (probably not significant) was more common in PLwHIV. Given this is probably the most novel piece of data in this study it would be nice if it were slightly better presented.
5. oropharyngeal swabs. Page 4 and 10 states oropharyngeal swabs were utilized all other references suggest these were nasopharyngeal swabs. Likely this is a typo to correct. Suspect they all should be nasopharyngeal given it was a PCR assay.
6. A comment is made that tabulation of “febrile” illness rate is possible and likely it would be worthy to comment how this related to the subsets of acute illnesses and viral recovery from nasopharyngeal swabs.
7. The one factor that may be overlooked is the comfort with which PLwHIV may feel in interacting with health care professionals that may differ from the control population. It is possible that there is a bias towards reporting symptoms in this population given ongoing drug side effects and discussion of them not only establish rapport with health care professionals but may also have resulted in beneficial interactions. There seems to be proof of this in that the use of over the counter medications was equivalent despite the fact that more contact with providers and actual prescribed medications was higher in PLwHIV. Whether this is conditioned by health care providers or as stated a manifestation of mild COPD and PRISm would be nearly impossible to separate as finding a control population with a similar degree of health care exposure would be impossible.
